# Chromium and formoterol therapy for obesity-induced asthma in rats

**DOI:** 10.3389/fphar.2025.1537022

**Published:** 2025-04-02

**Authors:** Rania T. Ibrahim, Yasser M. Moustafa, Maha Abdullah Alwaili, Amjad N. Alrebdi, Afaf Alharthi, Noha R. Noufal, Dina M. Khodeer

**Affiliations:** ^1^ Department of Scientific Research, Egypt Healthcare Authority, Ismailia, Egypt; ^2^ Pharmacology and Toxicology Department, Faculty of Pharmacy, Badr University in Cairo (BUC), Badr City, Cairo, Egypt, Egypt; ^3^ Department of Pharmacology and Toxicology, Faculty of Pharmacy, Suez Canal University, Ismailia, Egypt; ^4^ Department of Biology, College of Science, Princess Nourah bint Abdulrahman University, Riyadh, Saudi Arabia; ^5^ Department of Clinical Laboratory Sciences, College of Applied Medical Sciences, Taif University, Taif, Saudi Arabia; ^6^ Basic Medical Science Department, College of Medicine, Dar Al Uloom University, Riyadh, Saudi Arabia; ^7^ Pathology Department, Faculty of Medicine, Suez Canal University, Ismailia, Egypt

**Keywords:** chromium, formoterol, asthma, obesity, inflammatory markers, lipid profile

## Abstract

The development of asthma is impacted by fat. Asthma is more common in obese persons. The purpose of the experimental study is to determine how chromium, formoterol, and their combination can improve the quality of life for obese people with lung anomalies. Thirty-six male Wistar rats were divided into six groups: control (C), obesity (CO), obese-asthma (COA), and obese-asthma groups treated with formoterol (OAF), chromium (OACR), or both (OACRF). Except for group C, all groups received a high-fat diet for 4 weeks. Subsequently, ovalbumin (OVA) was administered subcutaneously (s.c.) to all groups except C and CO to induce sensitization. Asthma was triggered via 1% OVA aerosol challenges on days 26–28. Over 5 days, OAF and OACRF received daily formoterol inhalations (50 μg/kg), while OACR and OACRF were given chromium (400 μg/kg). Treatments were timed to align with asthma induction protocols. Lipid profile and inflammatory indicators were examined at the end of the trial—Immunohistochemical analysis of lung tissue, Histopathological and lung tissue stained with Hematoxylin and Eosin. The combination therapy (OACRF) significantly reduced body weight (p < 0.05), lowered LDL and triglycerides, increased HDL, and normalized lung tissue architecture compared to controls. Immunohistochemistry revealed reduced IL-1β and IL-17α expression. The (OACRF) group demonstrated superior asthma control by reducing body weight, improving inflammatory indicators, and restoring lung tissue to its normal state by administering chromium and formoterol therapy. The most effective strategy for treating both obesity and asthma is to address their two connected conditions. These findings demonstrate that combined chromium and formoterol therapy effectively addresses metabolic and inflammatory components of obesity-induced asthma, offering a promising dual-target therapeutic strategy.

## 1 Introduction

In the contemporary medical literature, obesity is the accumulation of excessive adipose tissue in the abdominal region as visceral fat, a condition associated with many health risks (Magdy et al., 2020; Mohamed et al., 2021; Piché et al., 2020). This pathological state is implicated in the etiology of various chronic diseases, including but not limited to cardiovascular disease, type 2 diabetes mellitus, hypertension, chronic kidney disease, and a spectrum of malignancies. Furthermore, obesity and metabolic syndrome are known to predispose individuals to several pulmonary disorders, with asthma being a notable example (Abo-Al-Ela et al., 2014; Badawy et al., 2021a; Petrie et al., 2018). Globally, the prevalence of overweight individuals is estimated at approximately 13%, while the incidence of obesity among children and adolescents was reported at 17% for the period between 2011 and 2014 (AlEnazi et al., 2023; Shaban et al., 2023).

Moreover, the epidemiological trends indicate a concerning rise in global obesity prevalence, underscoring the urgent need for comprehensive strategies targeting its prevention and management (Hegab et al., 2018; Koliaki et al., 2023). The multifaceted etiology of obesity encompasses genetic, environmental, and behavioral factors, contributing to its complexity and the challenges inherent in its mitigation (Elmetwalli et al., 2023; Tiwari and Balasundaram, 2024). The socioeconomic implications of obesity are profound, encompassing increased healthcare costs, lost productivity, and diminished quality of life. Addressing this public health crisis necessitates a multidisciplinary approach, integrating individual, community, and policy interventions (Anekwe et al., 2020; Badawy et al., 2021b). Research efforts continue to explore innovative therapeutic and preventive measures, aiming to curb the rising prevalence of obesity and its associated comorbidities. As the understanding of obesity’s pathophysiology evolves, so does the potential for developing targeted interventions that address the underlying causes and risk factors, offering hope for more effective management and prevention strategies (El-Demerdash et al., 2021; Lin and Li, 2021; Smith et al., 2020).

Asthma, a chronic respiratory condition, manifests through a constellation of common symptoms such as wheezing, airway constriction, chest tightness, persistent coughing, and difficulties in breathing (Ukena et al., 2008; Zedan et al., 2021). These symptoms indicate the inflammatory processes occurring within the pulmonary airways, which can be precipitated by various triggers, including physical exertion, exposure to airborne irritants, meteorological variations, and viral respiratory pathogens (Attia et al., 2020; D’Amato et al., 2018). In the context of obesity, the pathophysiological landscape of asthma undergoes notable alterations; obese individuals exhibit an increased production of pro-inflammatory markers and a disrupted lipid metabolism within the pulmonary system (Elkelish, 2024; Khodeer, 2024; Shailesh and Janahi, 2022). This alteration in the inflammatory and metabolic milieu complicates asthma management, particularly given the diminished efficacy of corticosteroids in targeting the underlying metabolic and immunological dysfunctions characteristic of obesity-induced asthma (Forno et al., 2011; Peters et al., 2018). Consequently, the management of asthma in the context of obesity demands a tailored approach, focusing on both the respiratory and metabolic aspects of the condition. Current therapies for obesity-induced asthma, such as corticosteroids, show reduced efficacy in obese populations due to altered immune-metabolic interactions. Despite evidence linking adipokines and inflammation to asthma severity, no studies have explored the combined use of chromium (metabolic modulator) and formoterol (bronchodilator) to address this dual pathology.

Emerging evidence suggests that the interplay between obesity and asthma exacerbates the severity and control of asthma symptoms, necessitating alternative therapeutic strategies that address the unique pathophysiological features of asthma in obese patients (Garg et al., 2024). These strategies may include weight management programs, targeted pharmacotherapy that considers the altered response to conventional asthma medications, and interventions to modulate the inflammatory profile specific to obese individuals (Tchang et al., 2000). Understanding the intricate relationship between obesity and asthma is crucial for developing effective treatment plans that improve patient outcomes and quality of life. Further research into the mechanisms linking obesity with asthma is essential for identifying potential therapeutic targets and optimizing care for this subset of patients (Marko and Pawliczak, 2018a).

One endocrine organ that becomes bigger when overweight is adipose tissue. It produces inflammatory cytokines known as adipocytokinases (Hui et al., 2018). Examples of adipocytokinases are leptin and interleukin-6 (IL-6). There is some evidence that these adipocytokinases contribute to developing lung abnormalities and may cause subtle inflammation (Palma et al., 2022a). For example, a rise in leptin levels may modulate lung damage and repairs, leading to weight gain associated with asthma. In addition, tumor necrosis factor-alpha (TNF alpha) and interleukin-6 (IL-6) affected the progression of lung diseases (Wang and Hu, 2022a). Dipeptidyl peptidase-4 and sterol regulatory element binding proteins (SREBPs) contribute to obesity (DPP-4). One of the transcription factors that regulate lipid synthesis is SREBPS. Two categories, (SREPs1) and (SREPs2), were established for it. One subunit of SREBPs1, SREBPs1a, is responsible for developing and synthesizing lipids globally (Lee et al., 2017). The regulation of energy and fatty acid storage is overseen by (SREBPs1c). Many cell types, including immune cells, express (DPP-4). It is involved in immune response and inflammatory pathogenesis and is classified into many chemokines and peptide hormones (Huang et al., 2022).

To enhance the quality of life for patients experiencing obesity-induced asthma, they must receive specialized therapeutic interventions. Chromium picolinate, a dietary supplement, is pivotal in regulating carbohydrate and lipid metabolism, influencing the body’s lipid profile. Its significance extends to the modulation of lipid metabolism, which contributes to a reduction in total cholesterol levels and aids in reducing body fat accumulation (Ngala et al., 2018). This dual action of chromium picolinate could potentially offer therapeutic benefits in the management of obesity-induced asthma by addressing the metabolic dysfunctions that exacerbate asthma symptoms in obese individuals. Consequently, incorporating chromium picolinate into the treatment regimen for patients with obesity-induced asthma may represent a novel approach to mitigate the impact of metabolic factors on respiratory health, thus promoting an improvement in both metabolic and respiratory outcomes (Yuan et al., 2018a). Further research into the efficacy and safety of chromium picolinate supplementation in this patient population is warranted to elucidate its therapeutic potential fully.

Formoterol, a long-acting beta-agonist (LABA), is increasingly recognized for its efficacy in asthma management, offering a significant therapeutic option for patients requiring maintenance treatment (Rajesh et al., 2020). As a bronchodilator, formoterol operates by stimulating beta-2 adrenergic receptors in the airway smooth muscle, leading to relaxation and subsequent relief of bronchoconstriction. This mechanism of action provides a rapid onset of relief from asthma symptoms and sustained improvement in airway function over time (Reddel et al., 2023). Formoterol is distinct in its ability to commence action within minutes, and its effects can last for approximately 12 h, making it an effective agent for both the prevention of asthma symptoms and the control of exacerbations (Tashkin, 2020).

Incorporating formoterol into asthma management plans is predicated on its capacity to enhance lung function, reduce the frequency of asthma attacks, and improve the overall quality of life for individuals with asthma. It is often prescribed with inhaled corticosteroids (ICS), which address the underlying inflammation associated with asthma, offering a comprehensive approach to asthma management (Rupani et al., 2021). This synergistic combination has been shown to provide superior control of asthma symptoms and reduce the risk of exacerbations compared to the use of either medication alone.

This study’s primary aim is to investigate chromium supplementation’s efficacy in conjunction with formoterol therapy as a novel approach to managing asthma symptoms in overweight patients. Specifically, the study seeks to determine whether this combined intervention can serve as an effective strategy for obesity prevention, thereby improving the quality of life for patients with asthma exacerbated by overweight conditions. This research hypothesizes that the metabolic benefits of chromium, including its potential to modulate lipid metabolism and reduce body fat accumulation, when used alongside the bronchodilatory effects of formoterol, a beta 2 agonist, may synergistically alleviate asthma symptoms. The investigation aims to provide empirical evidence to support or refute the hypothesis that this combined therapeutic approach can significantly improve respiratory function, asthma symptom control, and overall patient wellbeing.

## 2 Materials and methods

### 2.1 Animals

The National Center of Research in Cairo, Egypt, provided the 36 male Wister rats utilized in this investigation; their weight ranged from 180 to 200 g. The rats were kept at about 22°C in stainless steel cages with a regular light-dark cycle. A high-fat diet (HFD) it was prepared by 6% fat, 8% water, 18% protein, and 5% fiber (Pala et al., 2020; Yuan et al., 2018), given to thirty rats. Every week, the body weights of the rats were noted. Approval was obtained from the Animal Ethics Committee of the Faculty of Pharmacy, Suez Canal University, Egypt (IACUC License No. 202011MA1). At the end of the study, 0% mortality was observed in the normal and obese-asthma groups.

### 2.2 Drugs and chemicals

The following reagents were obtained: ovalbumin (OVA), phosphate-buffered saline, and dimethyl sulfoxide (DMSO) were sourced from Sigma (St. Louis, United States). Sodium chloride was procured from ADWIC CO., located in Egypt. Formoterol was purchased from NOVARTIS PHARMA SAE in Cairo. Additionally, chromium was acquired from MEPACO-MEDIFOOD, an Egyptian company specializing in pharmaceuticals and medicinal plants.

### 2.3 Induction of asthma

All experimental groups, except for groups 1 and 2, were administered subcutaneous (S.C) injections of ovalbumin (OVA). To induce asthma in the animal model, additional treatments with 1% (OVA) aerosol were administered on days 26, 27, and 28 following the sensitization phase (Conrad et al., 2009).

### 2.4 Experimental design

The 36 rats were randomly divided into six groups, six rats per each.

Group (control (C)): Rats fed with Basal diet; Group 2 (control obese (CO)): Rats fed with HFD for 4 weeks (Pala et al., 2020; Yuan et al., 2018). Group 3 (control obese and asthma (COA)): Rats fed with HFD for 4 weeks. OVA for 28 days (Conrad et al., 2009). Group 4 (Obese and asthma with chromium picolinate (OACR)): Rats fed with HFD for 4 weeks. OVA for 28 days. Then, it was switched to a basal diet and treated with chromium picolinate 400 µg/kg of diet, which was dissolved in water and taken orally for 6 weeks. Group 5 (Obese and asthma with formoterol (OAF)): Rats fed with HFD for 4 weeks. OVA for 28 days. Then, switch to a basal diet. Then, treated with 50 µg/kg by inhalation of formoterol for 15 min once/day For 5 days, and the final dosage should be administered five to 6 hours (Showalter et al., 2018). Group 6 (Obese and asthma with chromium picolinate and formoterol (OACRF)): Rats fed with HFD for 4 weeks. OVA for 28 days. Then, switch to a basal diet. Then, treated with chromium picolinate elemental 400 µg/kg of diet was dissolved in water, after that 50 µg/kg by inhalation for 15 min once/day for formoterol (Supplementary Figure S1).

### 2.5 Sample collection and determination of body weight change

After 6 weeks of treatment and the end of the experiment, rats were sacrificed by cervical decapitation (Clarkson et al., 2022) under ketamine anesthesia. Blood samples were collected and centrifuged at 3,000 rpm for 15 min; serum was aliquoted and stored at −80°C for kits measurements. The lungs were rapidly dissected, weighted, and stored in 4% phosphate-buffered formalin for histological examination. The weight of the rats was measured and recorded each week during the experimental period.

### 2.6 Biochemical analysis

#### 2.6.1 Determination of lipid profile including serum total cholesterol, total triglyceride, LDL and HDL

Lipid profile including serum total cholesterol, total triglyceride, LDL and HDL was measured by Elisa kit (XPRESSBIO PRODUCTS).

#### 2.6.2 Determination of serum adiponectin (ADP) and leptin (LEP)

Quantitative detection of rat Adiponectin in cell culture supernatants using a sandwich ELISA kit (PicoKine^®^, United States). Leptin was measured by rat leptin ELISA kit (RayBio^®^, United States) using an automated ELISA reader. Absorbance (OD) was measured at 450 nm using a BioTek Synergy H1 reader.

#### 2.6.3 Determination of lung tissue level for nuclear factor kappa B (NF-kB), TNFα and toll-like receptor 4 (TLR4)

For the *in vitro* quantitative evaluation of nuclear factor kappa B (NF-kB) in rat tissue homogenates, a sandwich enzyme immunoassay was used (My BioSource, United States). TNFα was measured by quantitative detection of TNFα in rat cell culture supernatants ELISA kit (MY BioSource, United States). The quantitative assessment of TLR4 in cell culture supernates was performed *in vitro* using a sandwich enzyme immunoassay (My BioSource, United States).

### 2.7 Lung tissue histopathological investigations

Tissue Fixation: Lung tissues were immediately fixed in 10% phosphate-buffered formalin for 24–48 h to preserve cellular architecture. Embedding and Sectioning: Fixed tissues were dehydrated, cleared, and embedded in paraffin. Serial sections (2-μm thickness) were cut using a microtome for downstream analysis. Staining Protocol: Sections were stained with hematoxylin and eosin (H&E) using standardized protocols (Bancroft and Cook, 1994) to visualize general histology, including inflammatory infiltrates and structural alterations.

Histopathological Analysis: Fibrosis Assessment: Collagen deposition and fibrotic changes were evaluated using established diagnostic criteria (Cheng et al., 2021). Inflammatory Infiltration: Bronchial and alveolar immune cell infiltration (e.g., neutrophils, lymphocytes) was quantified to assess chronic inflammation (Zainal et al., 2019). Microscopic Evaluation: Five random fields per section were analyzed under light microscopy (10×–4×0 magnification) (Garlisi et al., 1997). Blinded scoring was performed by two independent investigators to ensure reproducibility.

### 2.8 Immunohistochemical analysis for IL-1β and IL-17α

Generally, tissue samples fixed in paraffin were regularly dewaxed, rehydrated, and rinsed in 3% hydrogen peroxide to inhibit endogenous peroxidase. Immunostaining was performed on 4-mm thick, deparaffinized slices and heated for 15 min in a 0.01 M citrate buffer solution (PH = 7) to extract antigen. Mice monoclonal antibodies against interleukin-17 alpha (IL-17α) and interleukin-1beta (IL-1β) were then applied to the sections for an entire overnight (BIO SB, Santa Felicia, USA, Cat. # CA93117). *Sections were incubated with HRP-conjugated goat anti-mouse IgG (1:500, Abcam #ab6789) for 1 h. Color was developed with DAB (Vector Labs #SK-4100). Staining intensity was quantified using ImageJ (v1.53).*

### 2.9 Statistical analysis

The expression for each result is the mean ± standard error of the mean (SEM). Benferroni’s *post hoc* is performed after one-way repeated measures analysis of variance (ANOVA) has evaluated the data. The statistical package for social science, version 26, was used to analyze the data (SPSS software, SPSS Inc., Chicago, USA). A statistically significant difference in means has been defined as P < 0.05.

## 3 Results

### 3.1 Effect of chromium and/or formoterol on % of body weight change in different treated groups

**FIGURE 1:**
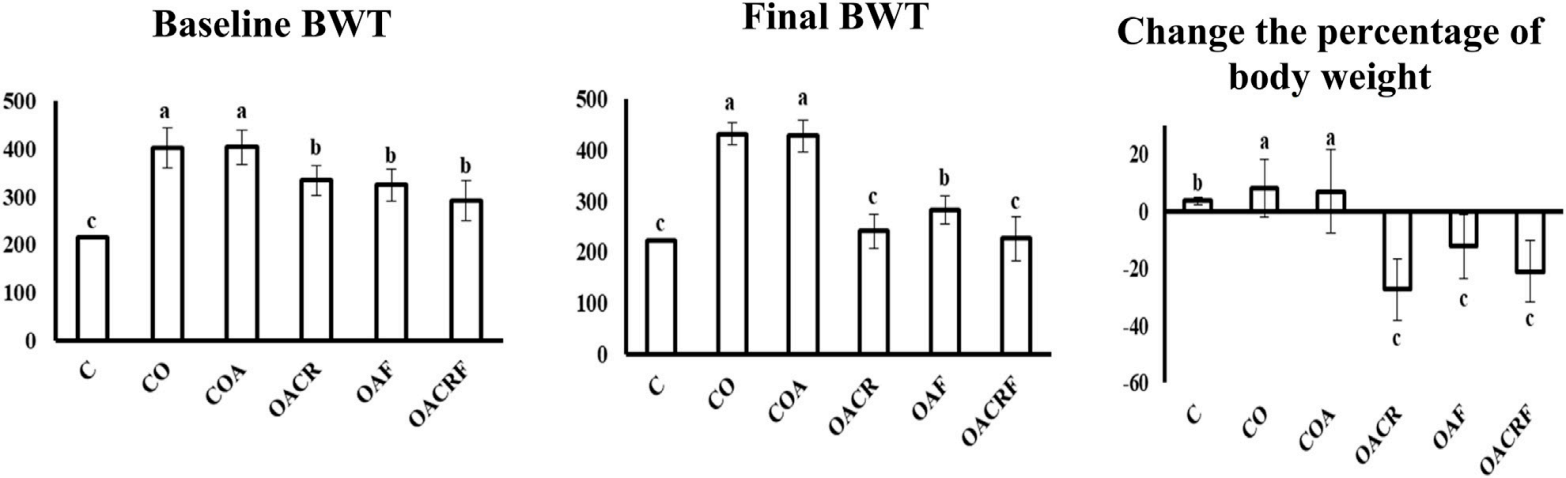
Effect of chromium picolinate on the percentage of body weight in different treated groups. Results are expressed as mean ± S. D and analyzed using one-way ANOVA followed by Bonferroni’s *post-hoc* test for multiple comparisons. C, control; CO, control obese; COA, control obese-asthmatic; OACR, obese-asthmatic treated with chromium picolinate; OAF, obese-asthmatic treated with formoterol; OACRF, obese-asthmatic treated with chromium picolinate & and formoterol. The different letters (a, b, and c) are above the bars mean a statistically significant difference between groups at *p* < 0.05, n = 6 3. The effect of chromium and/or formoterol on the level of Cholesterol, Triglyceride, HDL, and LDL in obese asthmatic rats.

Monitoring the body weight change during the experiment time, the data demonstrated a statistically significant difference in % body weight change across the four groups at P < 0.05 (Figure 1). The data representing a decrease in body weight percentage in the treated groups (OACR) and (OACRF) with chromium picolinate at a dose (400 μg/kg) compared to the (C) group. On the other hand, other groups (OAF), (CO), and (COA), which were not treated with chromium piclonate, did not show the noticed change in body weight percentage.

#### 3.1.1 The effect of chromium and/or formoterol on the level of Cholesterol, Triglyceride, HDL, and LDL in obese asthmatic rats

It was observed that there is a statistically significant difference between the four groups for Cholesterol, Triglycerides, and LDL at P < 0.05. It was revealed that there was a statistically significant difference between each group (Table 1). The data represents a decrease in the level of cholesterol in the group (OACR) and (OACRF), as well as a reduction in body weight percentage compared to (C) (Table 1). On the other hand, the other group (CO), (COA), and (OAF) recorded the highest value of the level of cholesterol according to its % of body weight (Figure 1; Table 1).

**TABLE 1:** Effect of chromium picolinate and formoterol on the level of lipid profile in serum including (A) total cholesterol, (B) total triglycerides, (C) LDL and (D) HDL. Results are expressed as mean ± S.E and analyzed using one way ANOVA followed by Bonferroni’s *post-hoc* test for multiple comparisons. C, control; CO, control obese; COA, control obese-asthmatic; OACR, obese-asthmatic treated with chromium picolinate; OAF, obese-asthmatic treated with formoterol; OACRF, obese-asthmatic treated with chromium picolinate & and formoterol. The different letters (a, b, c, d, e, and f) are above the bars, meaning statistically significant difference between groups at P < 0.05, n = 6.

	Mean	Std. Deviation	%Change	F	*p* value < 0.05
(A) Effect of chromium picolinate and formoterol on the level of Cholesterol					
C	42.6f	1.5		3,046.985	<0.001**
CO	136.8c	2.2			
COA	177.3a	2.2	316.2		
OACR	88.4d	2.6	107.5		
OAF	141.2b	1.3	231.5		
OACRF	51.8e	3.7	21.6		
(B) Effect of chromium piclonate and formoterol on the level of Triglycerides					
C	37.3f	2.2		2064.938	<0.001**
CO	153.4b	1.9			
COA	191.9a	2.0	414.5		
OACR	77.2d	2.2	107.0		
OAF	106.0c	3.7	184.2		
OACRF	54.4e	5.6	45.8		
(C) Effect of chromium picolinate and formoterol on the level of LDL					
C	16.6f	1.2		1,552.455	<0.001**
CO	92.0b	2.2			
COA	103.6a	2.3	524.1		
OACR	42.5d	1.9	156.0		
OAF	57.6c	3.1	247.0		
OACRF	32.7e	1.8	97.0		
(D) Effect of chromium piclonate and formoterol on the level of HDL					
C	32.9a	5.9		17.409	<0.001**
CO	22.3b	2.5			
COA	19.5c	3.5	−40.7		
OACR	30.1a	1.8	−8.5		
OAF	25.4b	2.0	−22.8		
OACRF	32.3a	1.8	−1.8		

**, means significant at P < 0.05, (−) means decrease according to C group.

According to the data (Tab ID), there is a statistically significant difference between the four groups for HDL at P < 0.05. It was revealed that there was a statistically significant difference between each group. The data showed increasing in the level of HDL in the group (OACRF) and (OACR), which decreased in % of body weight compared to (C) (Figure 1). The other groups (CO), (COA) and (OAF) showed the lowest value of the level of HDL according to its % of body weight (Table 1).

#### 3.1.2 The effect of chromium and/or formoterol on the serum level of adiponectin (ADP) and leptin (LEP) in obese asthmatic rats

**FIGURE 2:**
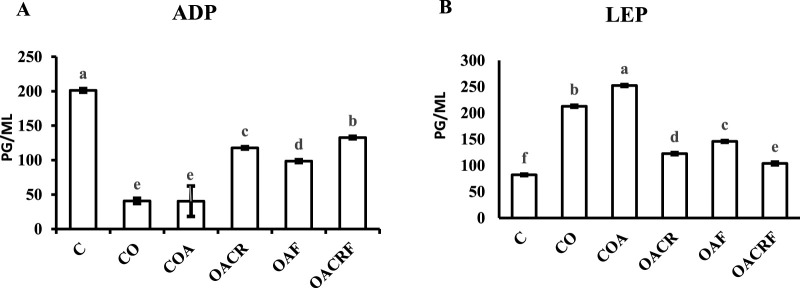
Effect of chromium picolinate and formoterol on lung tissue levels of **(A)** adiponectin (ADP), **(B)** leptin (LEP). Results are expressed as mean ± S.E and analyzed using one-way ANOVA followed by Bonferroni’s *post-hoc* test for multiple comparisons. C, control; CO, control obese; COA, control obese-asthmatic; OACR, obese-asthmatic treated with chromium picolinate; OAF, obese-asthmatic treated with formoterol; OACRF, obese-asthmatic treated with chromium picolinate & and formoterol. The different letters (a, b, c, d, e, and f) are above the bars, mean statistically significant difference groups at P < 0.05, n = 6.

**FIGURE 3:**
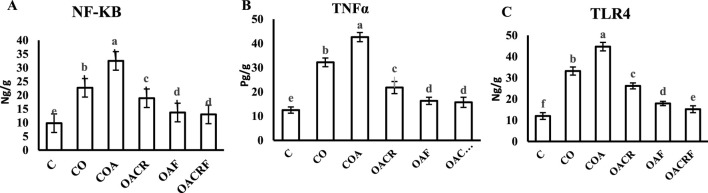
Effect of chromium picolinate and formoterol on lung tissue level of **(A)** NF-KB **(B)** TNF-α **(C)** TLR4. Results are expressed as mean ± S.E and analyzed using one-way ANOVA followed by Bonferroni’s *post-hoc* test for multiple comparisons. C, control; CO, control obese; COA, control obese-asthmatic; OACR, obese-asthmatic treated with chromium picolinate; OAF, obese-asthmatic treated with formoterol; OACRF, obese-asthmatic treated with chromium picolinate and and formoterol. The different letters (a, b, c, d, e, and f) are above the bars, mean significant difference between groups at P < 0.05, n = 6.

The data showed in (Figure 2A) a statistically significant difference between the four groups for ADP at P < 0.05. The data showed increasing in the serum level of ADP in the group (OACRF), (OACR), and (OAF), which decreased in % of body weight compared to (C) (Figure 1). The other group (CO) and (COA) showed the lowest value of the serum level of (ADP) according to its % of body weight (Figure 3A).

Regarding the data observed in (Figure 2B), there was a statistically significant difference between the four groups for LEP at P < 0.05. It was observed that there was a significant decrease in LEP serum level in treated groups (OACRF), (OACR), and (OAF) compared to (C) at P < 0.05. On the other hand, it showed the highest values in the percentage of body weight for the groups (CO) and (COA) (Figure 1).

#### 3.1.3 The effect of chromium and/or formoterol on lung tissue level of NF-kB, TNFαandTLR4 in obese asthmatic rats

**FIGURE 4:**
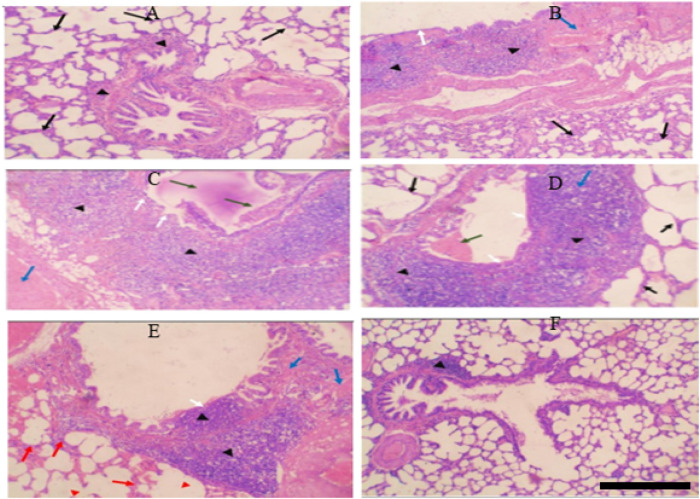
**(A)** Control group: Normal alveolar walls (black arrows) and bronchioles (black arrowheads). **(B)** Control-obese group: Fibrosis (blue arrow), chronic inflammation in bronchial walls (black arrowheads), thickened alveolar walls (black arrows), intact bronchiolar lining (white arrows). **(C)** Control-obese asthma group: Chronic inflammation (black arrowheads), fibrosis (blue arrow), mucus plug and obstructing cells (green arrows), bronchiolar epithelium ulceration (white arrows). **(D)** Chromium picolinate-treated group: Mild inflammation (black arrowheads), no fibrosis, small mucus plug (green arrow), intact bronchiolar lining (white arrow), normal alveolar walls (black arrows). **(E)** Formoterol-treated group: Reduced inflammation (black arrowheads), focal fibrosis (blue arrows), intact bronchiolar lining with focal ulceration (white arrow), alveolar hemorrhage (red arrows), emphysematous areas (red arrowheads). **(F)** Combined treatment: Restored lung tissue, minor lymphoid aggregate in bronchiolar walls (black arrowheads), no inflammation, fibrosis, or hemorrhage.

Using of chromium picolinate at dose (400 μg/kg) and inhalation of formoterol at (50 µg/kg) for 15 min once/day in groups (OACRF) and (OAF) showed a significant decrease in (NF-kB), (TNFα) and (TLR4) compared to (C) at P < 0.05 (Figures 4A–C). On the other hand, the groups (CO), (COA), and (OACR), which were not treated with formoterol, showed the highest value of (NF-kB), (TNFα), and (TLR4) (Figures 3A–C).

##### 3.1.3.1 The effect of chromium and/or formoterol on lung tissue morphology after staining with hematoxylin and eosin

Before being embedded in paraffin, the samples were fixed with 10% formalin. Sections of 3 µm in thickness were taken from each block, put on a glass slide, stained with hematoxylin and eosin (H&E), and then analyzed (Fig V). The transverse section (T.S) of the control group’s lung tissue revealed homogeneous bronchioles and alveolar walls (H&E, 10x) (Figure 4A).

T.S. revealed considerable fibrosis and infiltration of chronic inflammatory cells involving the bronchial walls in the lung tissue from the control obesity group. Is the lining of the bronchi intact? Chronic inflammatory cells invade and thicken the alveolar walls (H&E, 10x). Alveolar infiltration of proliferating, persistent inflammatory cells is substantial. There are spots with emphysema (Figure 4B).

T.S in lung tissue from the control obesity-asthma group showed significant chronic inflammatory cell infiltration involving bronchiolar walls and significant fibrosis. There is a thick mucus plug and spelled inflammatory cells obstructing the bronchiolar lumen. There is sloughing and ulceration of bronchiolar epithelium (H&E, 10x). Most chronic inflammatory cells infiltrate composed of eosinophils. There are emphysematous areas, and chronic inflammatory cells infiltrate involving alveolar walls (Figure 4C).

T.S. in lung tissue from the control obesity-asthma group treated with chromium showed a mild reduction in the severity of chronic inflammatory cell infiltrate involving bronchiolar walls. No fibrosis could be seen. A small mucous plug is seen in the bronchiolar lumen. The bronchiolar lining is intact. The alveolar walls show no evidence of thickening or inflammatory cell infiltration (H&E, 10x) (Figure 4D).

T.S. in lung tissue from the control obesity-asthma group treated with formoterol showed a significant reduction in the severity of chronic inflammatory cell infiltrate involving bronchiolar walls. Small fibrosis could be seen. No mucous plug or spelled inflammatory cells are seen in the bronchiolar lumen. The bronchiolar lining is intact except for focal ulceration above inflammatory cell infiltrates. The alveolar walls show evidence of hemorrhage with few chronic inflammatory cells. There are emphysematous areas (H&E, 10x) (Figure 4E).

T.S. in lung tissue from the control obesity-asthma group treated with formoterol and chromium showed almost complete restoration of lung tissue to its normal state, except for a small lymphoid aggregate within bronchiolar walls. No evidence of diffuse chronic inflammation, fibrosis, ulceration, or hemorrhage (H&E, 10 x). The focal lymphoid aggregates within the bronchiolar wall (Figure 4F).

#### 3.1.4 Immunohistochemically analysis for IL-1β and IL-17α in the tissue of lungs

**FIGURE 5:**
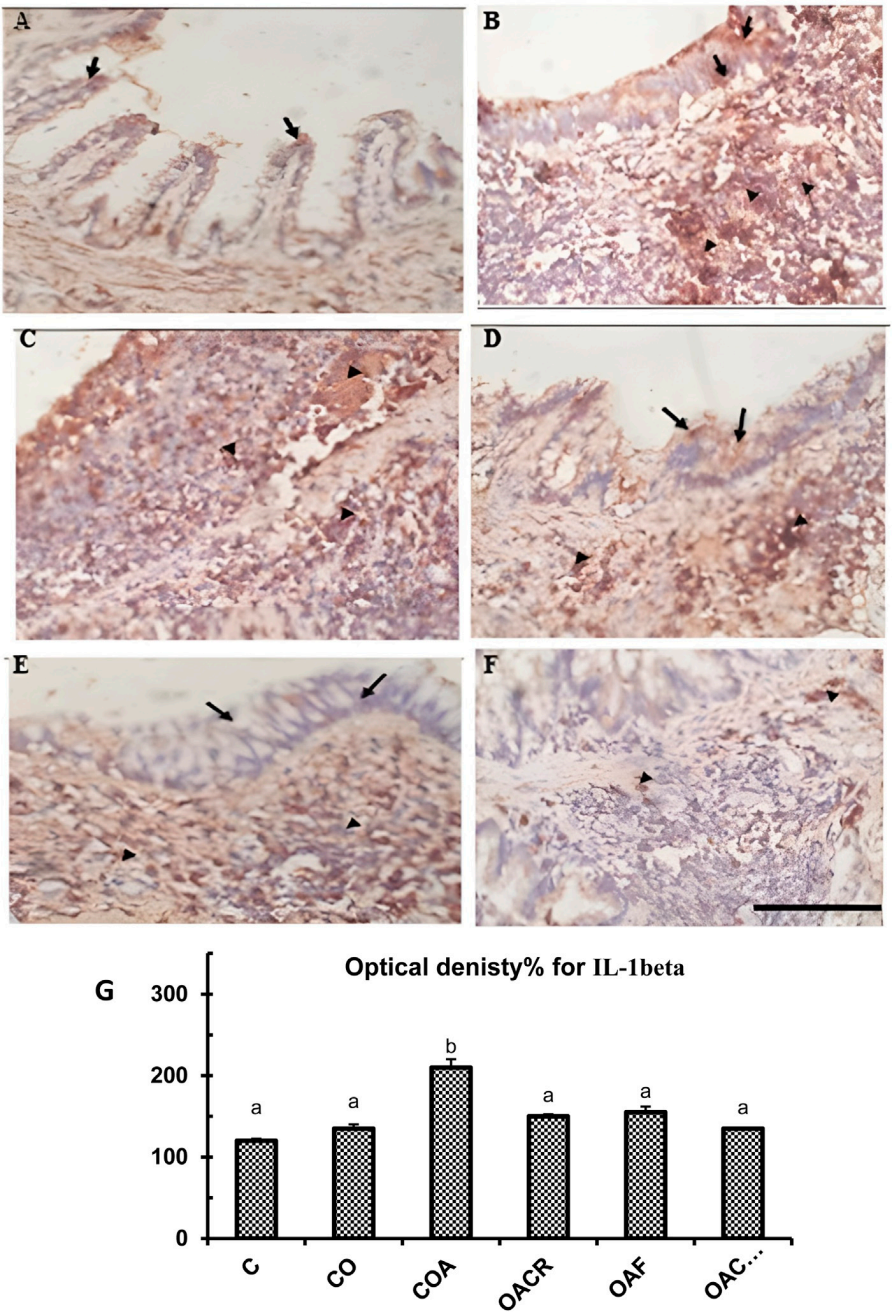
**(A)** Weak IL-1β expression in bronchiolar epithelial cells (black arrows) in control lung tissue (IHC, 40x). **(B)** Weak IL-1β expression in bronchiolar epithelial cells (black arrows) and moderate expression in chronic inflammatory cells (black arrows) in control obese lung tissue (IHC, 40x). **(C)** Strong IL-1β expression in chronic inflammatory cells (arrowheads) in control obese-asthmatic lung tissue (IHC, 40x). **(D)** Weak IL-1β expression in bronchiolar epithelial cells (black arrows) and chronic inflammatory cells (arrowheads) in asthmatic lungs treated with chromium picolinate (IHC, 40x). **(E)** Significant reduction in IL-1β expression with no bronchiolar epithelial cell expression (black arrows) and weak focal expression in chronic inflammatory cells (arrowheads) in obese-asthmatic lungs treated with formoterol (IHC, 40x). **(F)** Weak IL-1β expression in a few chronic inflammatory cells (arrowheads) in obese-asthmatic lungs treated with chromium picolinate and formoterol (IHC, 40x). **(G)** Optical density% of IL-1β expression in different groups expressed as mean ± S.E. Statistical analysis: one-way ANOVA with Bonferroni’s *post hoc* test, P < 0.05, n = 6. Groups: C, control; CO, control obese; COA, control obese-asthmatic; OACR, obese-asthmatic treated with chromium picolinate; OAF, obese-asthmatic treated with formoterol; OACRF, obese-asthmatic treated with chromium picolinate and formoterol. Different letters indicate significant differences between groups.

Tumor tissue was assessed. Positive cells were those that expressed IL-1β and IL-17α in the cytoplasm. Guidelines for a semi-quantitative grading system (Ilić et al., 2019). The staining intensity of the reaction (0–3) and the proportion of positive cells (0–5) were added to determine the final grades. The transverse section (T.S) of lung tissue from the control group revealed weak expression of IL-1β in a small number of bronchiolar epithelial cells (IHC, 40x) (Figure 5A). Furthermore, T.S. in lung tissue from the control-obese group revealed weak expression of IL-1β in bronchiolar epithelial cells with moderate expression of IL-1beta in chronic inflammatory cells (IHC, 40x) (Figure 5B). However, strong expressions of IL-1β were observed in chronic inflammatory cells (IHC, 40x) and lung tissue from the control obese-asthmatic group (Figure 5C). Further, there was a weak expression of IL-1β in bronchiolar epithelial cells and chronic inflammatory cells (IHC, 40x) (Figure 5D) observed in lung tissue from the obese-asthmatic group treated with chromium picolinate. Similarly, there was a significant decrease in IL-1β expression was seen in T.S. lung tissue from the formoterol-treated obese-asthmatic group in the epithelial cells of the bronchi (IHC, 40x) along with weak localized expression of IL-1β (Figure 5E). When both formoterol and chromium picolinate were given to the obese-asthmatic group, T.S. in their lung tissue revealed weak expression of IL-1β in a small number of chronic inflammatory cells (IHC, 40x) (Figure 5F).

**FIGURE 6:**
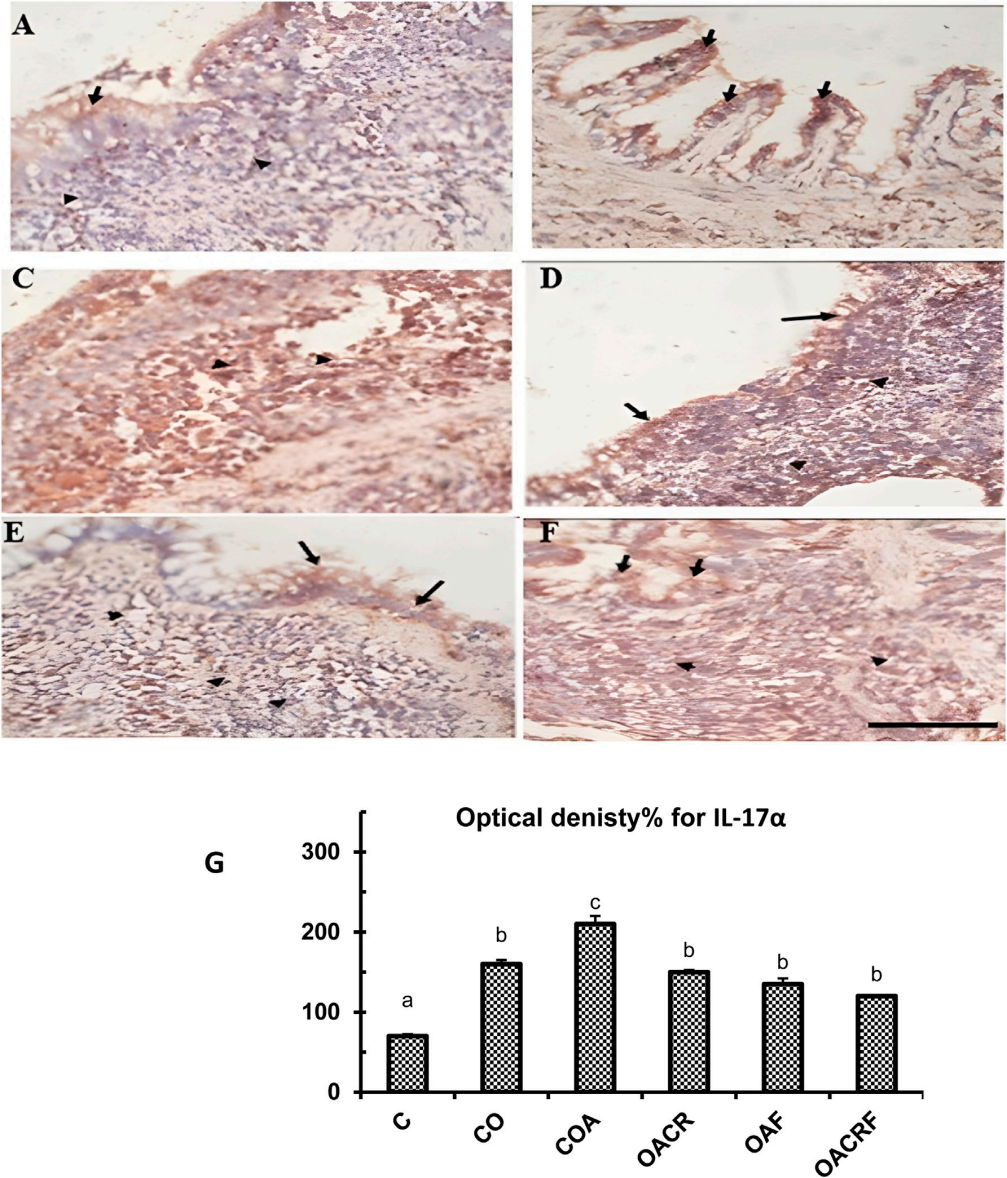
**(A)** T.S in lung tissue from the control group of IL-17α showed weak focal expression of IL-17α in a few bronchiolar epithelial cells (Black arrows) (IHC, 40x). **(B)** T.S. in lung tissue from the control obese group of IL-17 α showed moderate expression of IL-17α in bronchiolar epithelial cells (Black arrows) and in chronic inflammatory cells (Black arrows) (IHC, 40x). **(C)** T.S. in lung tissue from the control obese-asthmatic group of IL-17α showed Strong expression of IL-17α in chronic inflammatory cells (Arrow heads) (IHC, 40x). **(D)** T.S in lung tissue from obese-asthmatic group treated with chromium picolinate of IL-17α showed Moderate expression of IL-17α in bronchiolar epithelial cells (Black arrows) and in chronic inflammatory cells (Arrowheads) (IHC, 40x). **(E)** T.S in lung tissue from obese-asthmatic group treated with formoterol of IL-17 α showed Weak expression of IL-17α in bronchiolar epithelial cells (Black arrows) and in chronic inflammatory cells (Arrowheads) (IHC, 40x). **(F)** T.S in lung tissue from obese-asthmatic group treated with both formoterol and chromium picolinate of IL-17α showed Moderate expression of IL-17α in bronchiolar epithelial cells (Black arrows) and in chronic inflammatory cells (Arrowheads) (IHC, 40x). **(G)** Optical density% of expression for IL-17α in different groups. Results were expressed as mean ± S.E and analyzed using one-way ANOVA followed by Bonferroni’s *post-hoc* test for multiple comparisons. C, control; CO, control obese; COA, control obese-asthmatic; OACR, obese-asthmatic treated with chromium picolinate; OAF, obese-asthmatic treated with formoterol; OACRF, obese-asthmatic treated with chromium picolinate & and formoterol. The different letters (a, b, c, d, e, and f) are above the bars, mean significant difference between different groups at P < 0.05, n = 6.

On the other hand, The transverse section (T.S) of lung tissue from the control group revealed weak expression of IL-17α in a small number of bronchiolar epithelial cells (IHC, 40x) (Figure 6A). While T.S. in lung tissue from the control obese group revealed moderate focal expression of IL-17α in both bronchiolar epithelial cells and chronic inflammatory cells (IHC, 40x) (Figure 6B). Strong expressions of IL-17α were observed in chronic inflammatory cells (IHC, 40x) and lung tissue from the control obese-asthmatic group (Figure 6C). Further, moderate expression of IL-17α in bronchiolar epithelial cells and chronic inflammatory cells was observed in lung tissue from the obese-asthmatic group treated with chromium picolinate (IHC, 40x) (Figure 6D). Further, weak expression of IL-17α in bronchiolar epithelial cells and chronic inflammatory cells was observed in lung tissue from the obese-asthmatic group treated with formoterol (IHC, 40x) (Figure 6E). When both formoterol and chromium picolinate were given to the obese-asthmatic control group, T.S in their lung tissue revealed Prominent expression of IL-17α in bronchiolar epithelial cells and chronic inflammatory cells (IHC, 40x) (Figures 6F, G).

## 4 Discussion

This study aimed to investigate the protective effects of chromium and formoterol on experimentally induced asthma in obese rats. An asthmatic obese rat model was created by feeding rats a high-fat diet along with subcutaneous injections and inhalation of ovalbumin (OVA) to exacerbate asthma. The pilot trial successfully induced asthma in the rats. The primary objective was to evaluate the impact of chromium and formoterol on the asthma condition in these obese rats, with their weights recorded weekly. Studies on obesity and its metabolic consequences are crucial due to the close link between the immune system and metabolic pathways. Therefore, Signaling pathways that respond to diet and pathogens are highly integrated and evolutionarily conserved. Innate recognition receptors detect obesity-related foods, triggering pro-inflammatory and stress responses when consumed in excess (Rogero and Calder, 2018). Saturated fatty acids activate nuclear factor-kabba (NF-kB) and upregulate the pro-inflammatory cytokine tumor necrosis factor alpha via Toll-like receptors (TLR4) and (TLR2). This triggers the innate immune response (Salas-Venegas et al., 2022; Scott et al., 2011).

Likewise, In obesity, adipocytes secrete inflammatory mediators due to an adipokine imbalance. These mediators activate the inflammatory microsome, including NLRP3 and M1 macrophages. The release of IL-1β and IL-17α secreted by M1 macrophages in the lungs and adipose tissue is enhanced when NLRP3 is activated, alleviating asthmatic episodes in patients. Among the factors leading to asthma development include pro-inflammatory cytokines such as IL-1β and TNFα (Bantulà et al., 2021).

The study found that obesity and obese-asthma groups had significant weight changes, lower HDL, and higher total cholesterol, TG, and LDL compared to the normal group. This increased TNF-alpha and activation of inflammatory pathways (TLRs, IKKs, NF-KB). Additionally, These groups showed higher leptin and lower adiponectin levels. Obesity, characterized by excess fat storage, has been linked to health issues like asthma (Bonetti et al., 2022; Michelini et al., 2020). Body mass index (BMI) is the gold standard for determining excess body weight by indicating that being overweight is positively related to low HDL levels and high TG, LDL, and cholesterol levels (Ding et al., 2022). Previous study indicates that there is a negative correlation between body fat percentage and body mass index (BMI) and brown adipose tissue. Additionally, the adipose tissue of lean people primarily secretes anti-inflammatory indicators, whereas the adipose tissue of obese people secretes more pro-inflammatory markers (Akhter et al., 2021; Khanna et al., 2022). Furthermore, Immune cells called macrophages sense and respond to various stimuli in metabolic organs, such as adipose tissue, leading to persistent inflammatory reactions (Cai et al., 2018; Tabas and Lichtman, 2017). Inflammatory cytokines and chemokines, such as tumor necrosis factor-alpha, have important roles in inflammation initiation and resolution and are played by pattern recognition receptors, also known as toll-like receptors (Ben et al., 2019). NF-KB, a transcription factor involved in immune-mediated and inflammatory disorders, is activated by TNF receptor-associated factor 6 (TRAF6) (Yamamoto et al., 2021), activating IKKs (Kawai and Akira, 2010; Liang et al., 2022). Additionally, it is well-documented that circulating levels of pro-inflammatory cytokines grow in obese individuals due to excess adipose tissue (Palma et al., 2022; Scott et al., 2011). Adipocytes transmit information about the status of peripheral tissues' energy reserves to the brain via the hormone leptin. Leptin controls metabolism and food intake by sending signals to the brain’s control centers. Regarding metabolic diseases and obesity in humans, the leptin system’s network is crucial (Stover et al., 2023).

Furthermore, one component of the negative feedback loop that regulates body weight is the production and release of leptin by adipose tissue in fat. It enters the hypothalamus via the circulatory system and acts by binding to leptin receptors. The quantity of adipose tissue in your body is closely correlated with the level of leptin in your blood. Stated differently, a lower body fat percentage corresponds to a higher leptin level, and *vice versa* (Obradovic et al., 2021). Moreover, when plasma leptin levels drop, which activates orexigenic responses and represses anorexigenic responses, energy expenditure is suppressed, and food intake is raised until fat mass is recovered. As fat mass grows, the hypothalamic neural circuits work together to make people eat less and burn more calories. This increases plasma leptin levels (M.-D. Li, 2011; Martelli and Brooks, 2023).

Regarding the change in body weight percentage, the body secretes the hormone leptin, which aids in long-term weight maintenance (Izquierdo et al., 2019; Landecho et al., 2019). The hormone leptin controls hunger, weight, reproductive health, fetal development, and immunological responses that promote inflammation (Obradovic et al., 2021). Following caloric restriction, the concentration of circulating leptin falls, but it rises during refeeding (Hernández et al., 2000; Obradovic et al., 2021).

Additionally, adipose tissue is responsible for producing and secreting adiponectin. Unlike other adipokines, adiponectin levels are inversely related to total fat mass (Clemente-Suárez et al., 2023; Silha et al., 2003). This is especially true in the case of the adiponectin-leptin interaction. These two adipokines are controlled in opposing ways under nearly physiological circumstances. When leptin levels are high, it indicates that adiponectin is low, and *vice versa* when leptin levels are low. Furthermore, adiponectin secretion is regulated by adipose tissue quality, not quantity (Zhao et al., 2021).

On the other hand, asthma is only one of several allergy diseases linked to mast cells, which are innate immune cells (Shou et al., 2019). The stimulation of mast cells also initiates signaling pathways, including those involving NF-kB. These pathways produce inflammatory cytokines such as tumor necrosis factor-alpha (Lertnimitphun et al., 2021). OVA may bring on inflammation of the airways and asthma. Furthermore, increased TLR4 signaling might be involved (Wu et al., 2022).

Moreover, previous research has shown that obesity is one of the major pathogenic variables associated with asthma. The body mass index (BMI) measures a patient’s illness severity (Mohammed et al., 2023). There may be a connection between the pathophysiology of asthma and the adipocytes, adiponectin, and leptin produced by the body (Ma et al., 2019).

Our study found that chromium picolinate therapy significantly reduced body weight in obese-asthmatic subjects compared to controls. This weight loss improved lipid profiles by decreasing TG and LDL levels and increasing HDL levels. Chromium also reduced inflammatory cytokines and inhibited NF-kB pathways, lowering BMI. Asthma symptoms improved due to reduced fat cells, lower leptin, and higher adiponectin levels. Histopathological analysis showed reduced chronic inflammatory cell infiltration and no fibrosis in the chromium-treated group, while the control group had significant fibrosis and chronic inflammation. Immunohistochemically, the chromium-treated group had minimal IL-1β and moderate IL-17α expression compared to strong expressions in controls. Chromium therapy alleviated lung inflammation, reduced fibrosis, and improved respiratory health in obese-asthmatic subjects. Previous studies showed that chromium picolinate has been shown in earlier research to have the potential to prevent obesity (Pala et al., 2020). In lipid metabolism, chromium is an important player. Chromium is believed to influence several pathways that regulate hunger, energy balance, and caloric intake (Aksoy et al., 2021). Research has shown that chromium may help people lose weight while maintaining muscle mass. So, it is best used for slimming down (Derosa et al., 2019). It has been found that lowering body weight improves lipid profiles in overweight people. This included reducing triglycerides, raising HDL, decreasing LDL concentration, and reducing pro-inflammatory characteristics (Zhou et al., 2022). A healthier lipid profile (lower LDL-C, TG, and total cholesterol (TC) and higher HDL-C) was related to a significant reduction in body weight (Georgoulis et al., 2022). Regarding body weight, the previous research found that the risk of developing late-onset asthma was much higher when the body weight percentage rose. There is a correlation between obesity and an increased risk of developing asthma in people of all ages and genders (Nystad et al., 2004; Sikorska-Szaflik et al., 2022). Because of impaired lung function, obesity makes asthma management more challenging. Exacerbations are more common in obese asthmatics compared to those who are not overweight (Barros et al., 2017; Reyes Noriega et al., 2023). Furthermore, there is some overlap between the inflammatory processes that cause asthma and fat. This discovery suggests that the two inflammatory processes may contribute to the problem. Adipose tissue in obese asthmatics produces more pro-inflammatory cytokines, which may affect their lung function and clinical symptoms (Bantulà et al., 2021). Moreover, it is found that leptin mediated an indirect connection between obesity and chronic asthma (Z. Li et al., 2019). According to the research, asthma was also linked to elevated leptin levels. According to Bantulà et al., (2021), the leptin pathway might also explain the obesity-asthma connection. Elevated levels of interleukins, and TNF-α, are also linked to the obesity-related asthma phenotype (Leiria, Martins, and Saad, 2015; Wang and Hu, 2022). Asthmatic patients showed an increase in TNF-α expression proportional to their BMI (Tenório et al., 2021). Likewise, reducing body fat improves lung function tests, quality of life, and asthma control and exacerbations (Dias-Júnior et al., 2014; Eslick et al., 2020). By lowering the expression of target genes, such as those involved in inflammation (e.g., adiponectin, (TNF-α), leptin, and TLR 4), obesity treatment leads to the prevention of NF-kB activation (Kasprzak-Drozd et al., 2022). This research found that chromium may help lower body weight, which in turn helps regulate asthma by reducing inflammation (Fotschki et al., 2023).

Our study showed better improvement in lipid profile of OAF group than untreated group specifically in serum cholesterol, TG and LDL. Formoterol (50 μg/kg) inhaled for 15 minutes daily over 6 days alleviated asthma symptoms. The weight of the formoterol-treated obese-asthmatic group (OAF) was lower than the control groups (CO and COA) but higher than the chromium-treated groups (OACR and OACRF). OAF had better serum cholesterol, triglycerides, and LDL levels than CO and COA, but these levels were still higher than OACR and OACRF. HDL levels in OAF were similar to CO but differed from OACR and OACRF, indicating chromium’s superior impact on the lipid profile. TNF-α was significantly reduced in OAF compared to CO, COA, and OACR, with no significant difference between OAF and OACRF in TNF-α and NF-κB levels. Formoterol improved adiponectin and leptin levels, enhancing asthma conditions compared to control groups, with significant body mass index differences between OAF and chromium-treated groups. Chromium treatment also improved lung inflammation in the obese-asthma group compared to controls, which had substantial fibrosis, bronchiolar wall infiltration, chronic inflammatory cells, thick mucus plugs, and epithelial sloughing and ulceration. Additionally, prior research found that formoterol treatment reduced generated inflammatory mediators by enhancing the inflammatory marker, a consequence of the drug’s anti-inflammatory effects (Zhang et al., 2022).

Formoterol significantly reduced chronic inflammatory cell infiltration and mucus plugs in the bronchiolar walls of the OAF group. The CO group had severe fibrosis and inflammation, while the COA group showed extensive fibrosis, inflammation, and epithelial damage. Immunohistochemistry showed decreased IL-1β and IL-17α expression in the OAF group compared to the COA group. Moreover, With a short half-life (1–3 min) and a lengthy half-life (12 h), formoterol is unlike any other selective long-acting β2-agonist (Burkes and Panos, 2020; Rodrigo et al., 2010). Asthma management has been improved by using formoterol as maintenance medication or as required in mild, moderate, and severe chronic cases (Cusack et al., 2020; Pauwels et al., 2003). Reasons to use formoterol include its long half-life and the possibility that it might lessen the frequency of bronchodilator dosing. Formoterol, an inhaled long-acting beta2-agonist, is useful in managing chronic asthma because it improves lung function and decreases the frequency of asthma attacks (Reddel et al., 2022; Rodrigo et al., 2010). Treatment with formoterol decreases induced NF-kB activation and TNF production and has an anti-inflammatory effect (Martín et al., 2018). The pro-inflammatory adipokine adiponectin suppresses the cytokines nuclear factor-B and tumor necrosis factor-contributing to inflammation. Obese people have lower amounts of adiponectin, even though it is mostly produced by visceral adipose tissue. A possible explanation for this mystery might be the finding that macrophages produce TNF-alpha in adipose tissue, which could directly restrict the production of adiponectin (Kim et al., 2014; Zorena et al., 2020). The reason formoterol lowered TNF-alpha production, adiponectin levels rose, and leptin levels fell was explained. If leptin levels are low, adiponectin levels are high, and *vice versa* (Zhao et al., 2021).

The study found that combining formoterol and chromium picolinate effectively manages asthma and improves health in obese rats. Formoterol, administered at 50 μg/kg daily for 6 days, reduced inflammation by lowering TNF-α and deactivating NF-kB pathways. Chromium picolinate, taken orally at 400 μg/kg for 6 weeks, significantly aided weight loss and lipid profile improvement. The OACRF group, receiving both treatments, showed the best results in reducing body weight, improving lipid profiles, and lowering inflammatory mediators, outperforming other groups. Thus, this combination therapy is the most effective for managing asthma and enhancing overall health in obese rats. In addition, prior research has shown that formoterol enhances the inflammatory mediator, reducing inflammation; chromium’s weight-loss effects and its impact on lipid profiles are also noteworthy (Zhang et al., 2022). Dietary changes and rising rates of overweight and obesity have been linked in earlier research to an increase in asthma cases. There is a correlation between obesity and asthma that is associated with worse asthma control, more severe asthma episodes, and an increased risk of asthma exacerbation (Marko and Pawliczak, 2018). Among the subgroups of people with moderate to severe asthma, several recent studies have focused on those who are overweight. When compared to the non-obese asthma population, these people have higher exacerbations, more treatments, reduced lung function, and more difficult-to-control asthma. Treatment to manage both body mass index and asthma was shown to be the most effective method for controlling asthma in the obese-asthmatic group in the present investigation (Bantulà et al., 2021). This study used male rats only; future work should include females. The treatment duration (6 weeks) may not reflect long-term effects. Mechanistic insights into chromium-formoterol synergy remain to be explored.

## 5 Conclusion

The study investigated the potential protective effects of chromium and formoterol in mitigating asthma symptoms in obese rats. The combination of these therapies demonstrated significant improvements in metabolic and respiratory parameters, highlighting a promising strategy for managing obesity-induced asthma. The dual treatment with chromium and formoterol effectively reduced body weight, improved lipid profiles, and decreased inflammatory markers, restoring lung tissue to its normal state. This synergistic approach addresses both the metabolic dysfunctions and respiratory challenges associated with obesity-induced asthma, offering a comprehensive therapeutic strategy. Further research is warranted to explore this combined therapy’s clinical applications in humans and elucidate the underlying mechanisms contributing to its efficacy.

## Data Availability

The original contributions presented in the study are included in the article/Supplementary Material, further inquiries can be directed to the corresponding author.
