# Supplementary figures and images for: Chromium and formoterol therapy for obesity-induced asthma in rats
Source: Front Pharmacol. 2025 Apr 2;16:1537022. doi: 10.3389/fphar.2025.1537022 (PMC12000533; doi:10.3389/fphar.2025.1537022)

## Experimental design

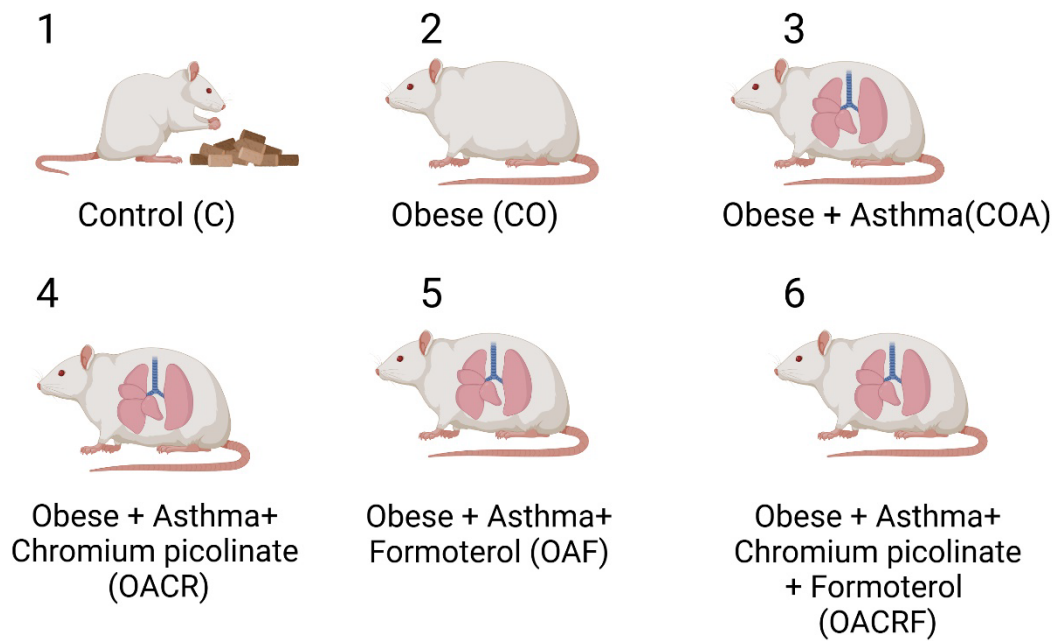

**Supplementary Figure 1: The Experimental design of the treatments**

Supplement: Supplementary file 1 [file Image1.pdf]
